# A novel method for alveolar bone grafting assessment in cleft lip and palate patients: cone-beam computed tomography evaluation

**DOI:** 10.1007/s00784-020-03505-z

**Published:** 2020-08-15

**Authors:** Marcin Stasiak, Anna Wojtaszek-Słomińska, Bogna Racka-Pilszak

**Affiliations:** Department of Orthodontics, Faculty of Medicine, Medical University of Gdańsk, Aleja Zwycięstwa 42c, 80-210 Gdańsk, Poland

**Keywords:** Alveolar bone grafting, Cleft lip, Cleft palate, Cone-beam computed tomography, Maxilla, Orthodontics, Retrospective studies

## Abstract

**Objectives:**

This retrospective cross-sectional study aimed to present a new method for secondary alveolar bone grafting (SABG) assessment and to qualitatively evaluate the SABG results in unilateral cleft lip and palate patients.

**Materials and methods:**

Research was conducted according to the STROBE guidelines. The study group consisted of 21 patients with a mean age of 16 years. High-resolution cone-beam computed tomography (CBCT) was performed at least 1 year after grafting. The experimental side was the cleft side, and the contralateral side without a congenital cleft was the control. Measurements were performed at four levels of the maxillary central incisors’ roots according to the new scale with scores from 0 to 3. The sum of the scores provided a general assessment of bone architecture. The Wilcoxon signed-rank test was used for intergroup comparisons, and a Kappa coefficient was used for reproducibility measurements.

**Results:**

High individual variability was found, and the bone architecture was significantly worse on the cleft side than on the noncleft side. The results showed 28.57% failure, 33.33% poor, 19.05% moderate, and 19.05% good results from the surgical procedure. Kappa coefficients produced results from 0.92 to 1.00 for intra-rater and from 0.81 to 1.00 for inter-rater reproducibility.

**Conclusions:**

CBCT provides detailed information about alveolar bone morphology. The new assessment method is useful at every treatment stage and provides excellent repeatability. SABG did not provide good bone morphology, in most cases.

**Clinical relevance:**

This research presents a new universal alternative for the assessment of SABG by utilizing CBCT.

**Electronic supplementary material:**

The online version of this article (10.1007/s00784-020-03505-z) contains supplementary material, which is available to authorized users.

## Introduction

Cleft lip and palate (CLP) is one of the most common congenital conditions in the facial segment of the cranium [1]. A characteristic feature of clefts includes partial or complete lack of anatomical tissue continuity and tissue hypoplasia in the affected area. Cleft is a developmental malformation resulting from both genetic and environmental factors [2].

The long-term treatment of CLP patients requires an interdisciplinary approach [3]. Rehabilitation protocol includes secondary alveolar bone grafting (SABG) that is performed when the patient presents with a mixed dentition [4]. The most favorable outcomes present when the lateral incisor or the canine erupts through the transplant and the bone are functionally loaded [5–9]. The purpose of the autogenous bone grafting is closure of the oronasal fistula and anatomical tissue continuity of the alveolar process in the maxilla [10].

Bone transplant results must be known to continue orthodontic treatment after the alveolar grafting. When a lateral incisor is missing (a frequent condition in CLP patients [11]), the results help specialists decide whether tooth replacement or space closure would produce the best outcome [12]. Orthodontic mesial movement of the posterior teeth to replace the missing incisor requires adequate position and volume of the bone bridge [13].

Currently, three-dimensional x-ray diagnostics provide an appropriate tool for the assessment of SABG treatment outcomes [12]. Since radiological protection is needed for this type of examination (especially in young patients), the use of cone-beam computed tomography (CBCT) over computed tomography (CT) examination appears justified [12, 14]. Furthermore, a small field of view (if there are no other indications for increasing the imaging area size) is recommended [12]. Adopted required follow-up period after SABG should be at least 1 year [12].

The best method for comparing the therapeutic effects of the procedure appears to be evaluation of the percentage value of the augmented or reconstructed bone defect supported by 3-D images. Preoperative and postoperative radiographs are necessary to evaluate these ratios [12], but two radiographs are not always available. Moreover, percentage ratios do not provide a spatial assessment of the bone bridge architecture. This deficiency presents a significant issue for further orthodontic treatment.

Interproximal alveolar bone height measurements on the root surfaces of the teeth adjacent to the cleft were widely used for the SABG assessment. The utilization of 2-D x-ray images and 2-D cross-sections from 3-D images provided this data. Two-dimensional vertical analysis or the assessment of the alveolar process position relative to the base of the nasal cavity is too simple to use as verification criteria for treatment outcome effectiveness. This limitation also makes it inappropriate for scheduling further orthodontic and prosthetic treatment [12].

The horizontal methods suggested by Wangsrimongikol et al. [15], Suomalainen et al. [16], and Garib et al. [17] seem suitable for the spatial assessment of the graft site and the areas of bone deficit. They may also be implemented when treatment modification is evaluated [12], but these methods have certain limitations. Wangsrimongkol et al. [15] used bone graft site width as the reference point, which could be difficult to precisely evaluate [12]. The grades presented by Suomalainen et al. and Garib et al. were evaluated in relation to the width of tooth roots. Suomalainen et al. [16] presented a method for early SABG results assessment. In line with permanent canine eruption, the reference points and correlated evaluation results change. This change presents a significant limitation for continuous assessment of the procedure in the same patient over longer follow-up periods. Garib et al. [17] described a method for late evaluation of the procedure after canine mesial movement into the graft area. This method could also be useful in cases without mesialization that still include completed canine eruption. However, early assessment before canine eruption is not feasible. Another limitation is excessive mesial angulation of the referential tooth after orthodontic space closure. This frequent presentation increases the risk of inappropriate assessment. Moreover, none of the aforementioned authors presented the generalization of the measurements to assess the surgical procedure’s final results.

For these reasons, this retrospective observational cross-sectional study designed a new method for SABG assessment.

The first aim of this study was to present a new three-dimensional method for SABG assessment. The next aim was to qualitatively evaluate the SABG results in unilateral cleft lip and palate (UCLP) patients treated in the same orthodontic department.

The null hypothesis was that the alveolar bone morphology is the same on the cleft and noncleft sides in UCLP patients after SABG.

## Materials and methods

Strengthening the Reporting of Observational Studies in Epidemiology (STROBE) guidelines were used in this study [18]. CLP patients are treated according to the complex protocol. For ethical reasons, it is not possible to obtain an untreated control group. Therefore, a split-mouth study design was selected. The experimental side was the cleft side, and control side was the contralateral side with normal anatomy. The study design was approved by the Ethics Committee of Medical University of Gdańsk (approval number NKBBN/311/2017).

The research was conducted in the Medical University of Gdańsk’s orthodontic department. The department has been utilizing 3-D x-ray imaging since 2017. CBCT, with a small field of view, is considered standard medical documentation in CLP patients. Patient qualification was performed from July 2018 to October 2018. There were two patients with operation histories in February 2005 and October 2006, respectively. The remainder was presented with operation histories from August 2011 to June 2017. Radiographs were taken from July 2017 to September 2018, and measurements were performed from June to October 2019.

Eligibility criteria were as follows: complete UCLP without other congenital deformities, SABG surgery, and CBCT imaging at least a year after grafting. Due to the inability to compare contralateral sites, bilateral clefts were not included in the study. Unilateral cleft lip and alveolus (UCLA) patients were also excluded due to qualitative reasons.

In the first stage of selection, all patients currently treated in the orthodontic department with complete UCLP recognition were identified using electronic medical records software: Estomed (Hakon Software, Gdańsk, Poland). Subsequently, patients were examined and qualified during their orthodontic appointments by all the authors. Analysis of the medical documentation was performed, and all patients who met the eligibility criteria were included in the study.

Study outcomes were qualitative measurements of the alveolar bone based on CBCT examination. Patients differed in their follow-up periods and orthodontic treatment stages. Potential confounders are artifacts due to metal-fixed orthodontic appliances [16].

The high-resolution CBCT examinations were performed with a CS 8100 3-D scanner (Carestream, Atlanta, USA). The imaging conditions were 80 kV, 5 mA, 12 s, voxel size of 0.2 mm, and field of view (FOV) of 5 cm × 5 cm. The images were analyzed by utilizing the CS 3-D Imaging Software (Carestream, Atlanta, USA).

**Fig. 1:**
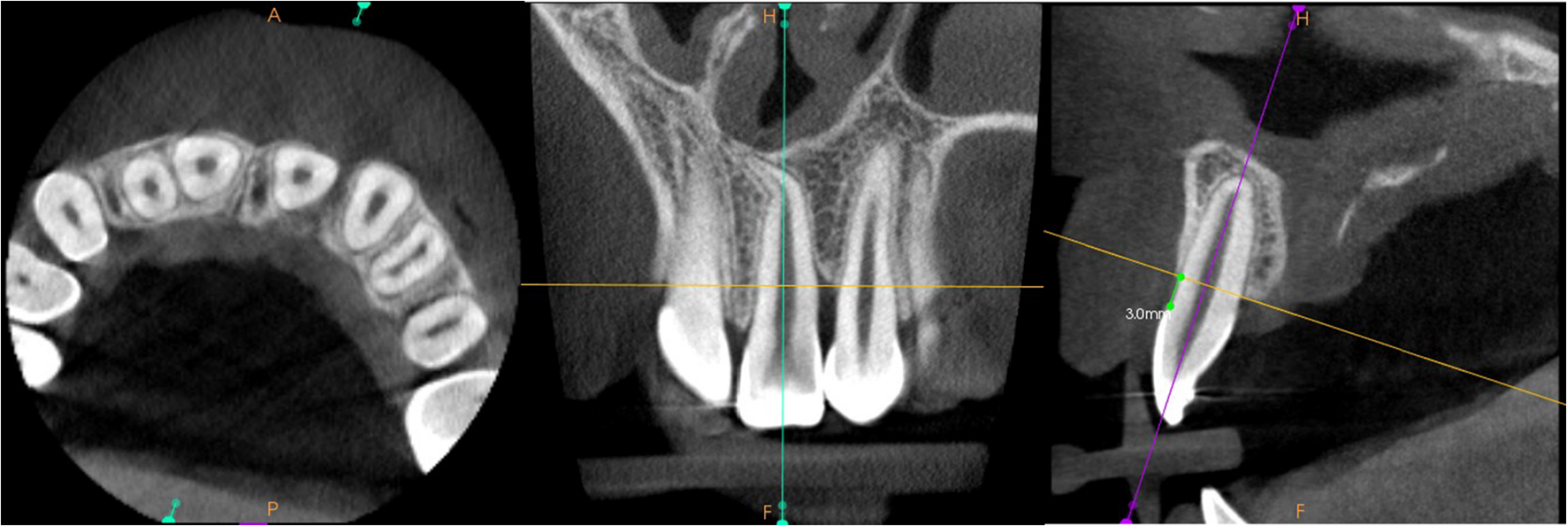
The CBCT images standardization

**Fig. 2:**
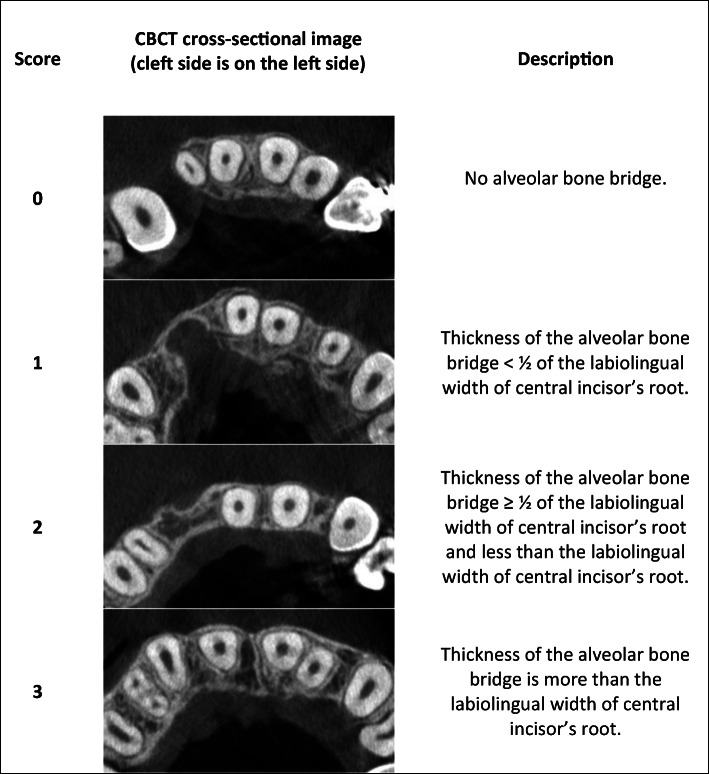
Horizontal scale

Standardization was obtained after reorientation of the images according to the long axes of central incisors on the corresponding side. The cementoenamel junctions were points of reference to establish the position of four assessment levels: 3 mm, 5 mm, 7 mm, and 9 mm. The cementoenamel junction point was set at the most apical point of the enamel on the incisor’s midsagittal cross-section (Fig. 1). The bone architecture was assessed according to the new method. In the first stage, an assessment of the presence or lack of the bone bridge due to the continuous investigation of the areas was conducted. In the next step, a classification of the bone was performed at the adequate levels in the narrowest points of the alveolar bone between canines and central incisors. This classification was made according to the new horizontal scale (Fig. 2). The final step involved summing all the scores on each side to obtain a general assessment of the bone architecture according to the interval scale: 0, failure; 1–4, poor results; 5–8, moderate results; and 9–12, good results. A total score of 0 was reserved for cases without any resulting bone bridge. There is a possibility that the narrow bone bridge is present but placed above or between the adopted measurement levels (3, 5, 7, or 9 mm). As a result, an assessment modification was elaborated for the cases with the bone bridge obtainment, which was not detected at any adopted measurement level. This evaluation was performed at the bone bridge level, and an adequate score was assigned to the nearest measurement level (3, 5, 7, or 9 mm). Moreover, in cases of severe central incisor root resorption, an assessment according to the horizontal scale was performed at the adequate level (9 mm) but with comparison with the root diameter measured 0.5 mm beneath the apex.

The total sample’s measurements were performed three times. The measurements were made twice within a 4-month interval by the first author and a month later by the second author. Both of the raters were trained orthodontists with experience in CLP cases.

Bias resulting from incisors tipping and angulation was eliminated by standardizing the reorientation of the images. By completing measurements three times, potential bias from inaccuracy was reduced.

Follow-up differences seem insignificant since the autogenous bone grafts show some stabilization after 1 year [6]. Moreover, on account of age, the influence of age-related periodontal atrophy could be discounted.

The database was collected in Microsoft Excel file (Microsoft, Washington, USA). Statistical analyses were performed with Statistica (version 13.3, TIBICO Software, Palo Alto, USA) and RStudio software (version 3.6.0, the R Foundation for Statistical Computing Platform, Boston, USA). Kappa correlation coefficient was used for intra-rater and inter-rater reproducibility measurements. Non-parametric Wilcoxon signed-rank test was used for the experimental side and control side comparisons. A bootstrapping analysis in RStudio was used for test power calculation.

## Results

In the first stage of selection, 62 patients were identified using electronic medical records software. Patients were excluded due to no bone grafting: before or not qualified (*n* = 31), primary bone grafting (*n* = 6), tertiary bone grafting (*n* = 2), and lack of CBCT examination with required follow-up period (*n* = 2). A group of 21 patients was confirmed eligible and further analyzed.

The study group consisted of 5 female (24%) and 16 male (76%) patients. There were 9 right (43%) and 12 left (57%) clefts. A lateral incisor was missing in 11 patients (52%). An equal number of patients had lateral incisors in the major and in the minor segments (*n* = 5). Cleft side canines were not fully erupted in two patients during the CBCT examination. Table 1 presents information about the sample.

**Table 1:** Characteristic of the study group

Characteristic	Mean (SD) (y)	Median (y)	Min-max (y)
SABG age	10.96 (1.81)	11.06	6.91–14.09
CBCT age	16.15 (2.84)	15.68	11.66–21.18
follow-up	5.19 (2.75)	5.34	1.18–12.43

SD, standard deviation; y, year; Min-max, minimum-maximum; SABG, secondary alveolar bone grafting; CBCT, cone-beam computed tomography

According to the Boyne and Sands technique, surgical operations took place in five rehabilitation centers and were performed by five plastic surgeons [4]. In our protocol, the bone graft surgeries were preferably performed between 7 and 11 years of age and when the permanent lateral incisors or the canines were covered by a thin bone layer. However, due to childhood diseases, orthodontic treatment process, or a lack of parental cooperation (non-compliance with the recommendations and treatment protocol), the surgeries were often performed at the later age. Preoperatively, the upper arch was expanded, and the teeth were aligned using fixed orthodontic appliances. Palatal expansion resulted in bone segment translocation, palatal soft tissue straining, and cleft space extension. The smaller the cleft gap is initially, the more favorable surgical conditions are at the outset. The cleft widths were evaluated individually during the surgery qualification processes. The primary limitations are the soft tissues and their low susceptibility to repositioning to reconstruct the continuity of the alveolus [10]. A tight socket from the full value soft tissues provides a good blood supply to the grafted cancellous bone and its stabilization. Oral cavity sanitation was performed before the alveolar grafting. Iliac crest bone grafts were used in 17 patients before canine eruption and in 4 patients before lateral incisor eruption. The surgeries were performed with antibiotic coverage (amoxicillin and clavulanic acid or clindamycin in the case of an allergy). Oral antibiotics began 1 h before the surgery and then continued for 5 days. Patients were asked to rinse their mouth with an antibacterial and antifungal fluid (Octenident, Schulke & Mayer), to brush their teeth the day after the surgery, and to have a semi-liquid diet for 4 weeks. The teeth adjacent to the cleft were cleaned with a soft postsurgical toothbrush and a low abrasive toothpaste. Orthodontic treatment was continued a month after the surgery.

**Fig. 3:**
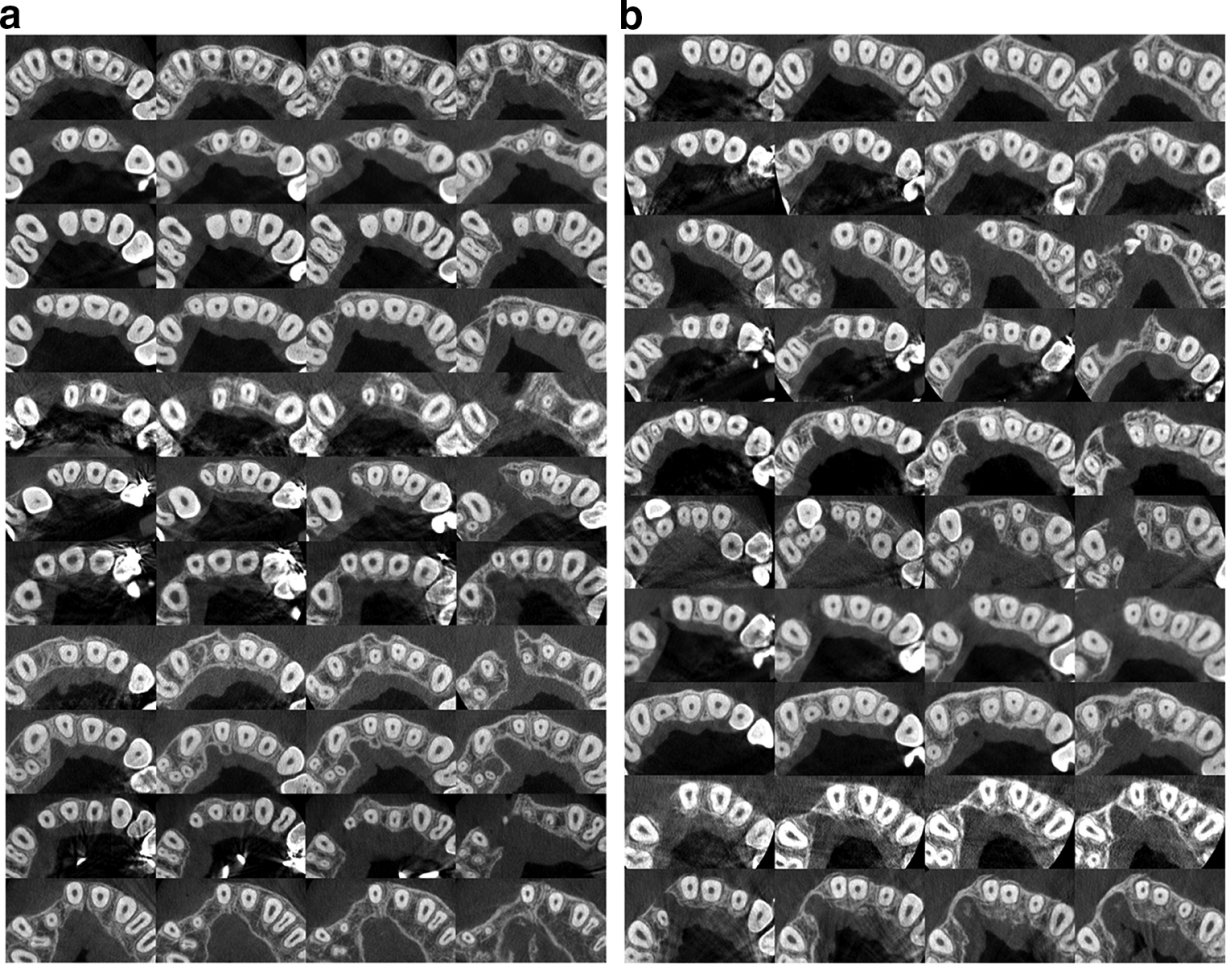
**a** and **b** Horizontal cross-sectional images for the assessment of the cleft side alveolar bone. Cleft sides are presented on the left. Each line represents one patient. Images are set from the measurement levels of 3 mm (left side) to these of 9 mm from the cementoenamel junctions (right side)

No metal artifacts preventing bone evaluation were noticed. The measurements on the cleft and noncleft sides were performed three times in all patients, and each of the measurement series included assessment of the 168 sites. Next, a consensus reading was performed by all the authors. On the cleft side, 39 sites were classified as 0, 19 sites as 1, 12 sites as 2, and 14 sites as 3 (Fig. 3a, b). On the control side, no 0 or 1 scores were obtained. There were 15 sites classified as 2 and 69 sites as 3 (Table 2). The narrow bone bridge, which was placed between measurement levels, was present in one patient. The measurement modification at the level of 9 mm was used in one patient due to the cleft side central incisor’s root resorption.

**Table 2:** Intergroup comparisons at different measurement levels

Measurement heights	Number of patients								*p*
	Cleft scores				Noncleft scores				
	0	1	2	3	0	1	2	3	
3 mm	13	3	3	2	0	0	10	11	0.000132*
5 mm	9	5	3	4	0	0	4	17	0.000438*
7 mm	7	7	2	5	0	0	1	20	0.000438*
9 mm	10	4	4	3	0	0	0	21	0.000196*

mm, millimeters; *Statistically significant at *p* < 0.05

**Fig. 4:**
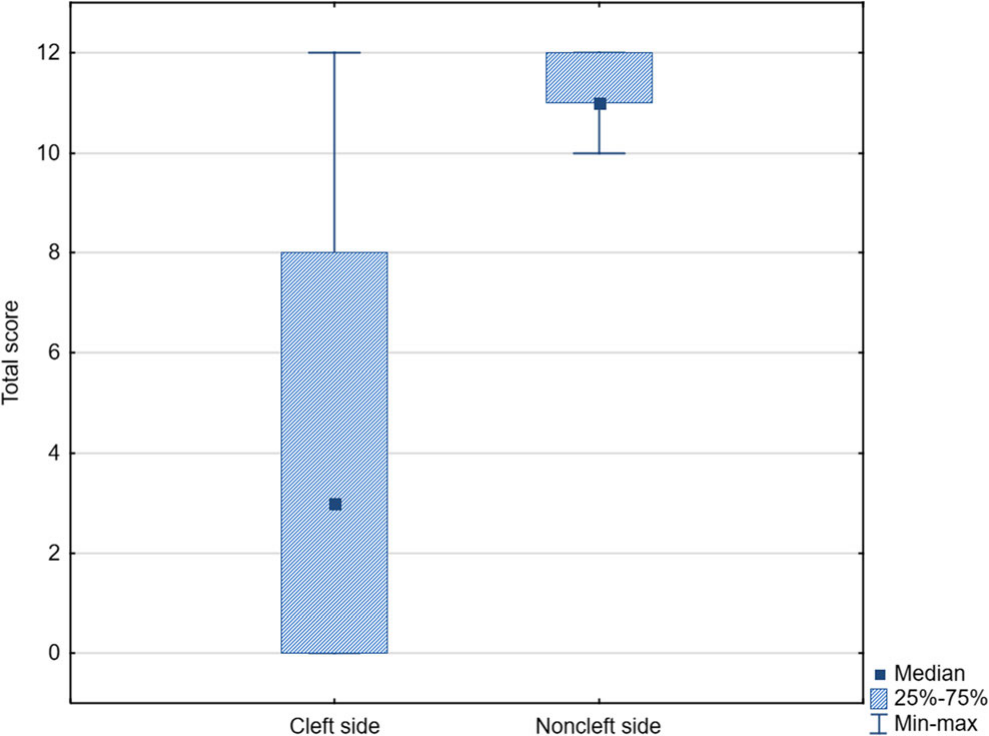
Box plot 25%-75% – 25-75 percentile, Min-max – Minimum-maximum

High individual variability was found (Fig. 4). The median total score was 3 on the cleft and 11 on the noncleft side. The results showed 28.57% failure, 33.33% poor, 19.05% moderate, and 19.05% good results of the surgical procedure. The alveolar bone was classified as good in all patients on the noncleft side (Table 3).

**Table 3:** Intergroup comparisons of the total score

Total score		Failure	Poor results				Moderate results				Good results				*p*
		0	1	2	3	4	5	6	7	8	9	10	11	12	
Number of patients	Cleft sides	6	3	0	3	1	2	0	0	2	1	0	2	1	0.000089*
	Noncleft sides	0	0	0	0	0	0	0	0	0	0	3	9	9	

*Statistically significant at *p* < 0.05

In all statistical measurements, a 95% confidence interval was adopted. Wilcoxon signed-rank test showed statistically significant (*p* < 0.05) differences between cleft and noncleft side measurements. The bone architecture was significantly worse on the cleft side than on the noncleft side for all measurement levels. Kappa coefficient results ranged from 0.92 to 1.00 for intra-rater and from 0.81 to 1.00 for inter-rater reproducibility. These results showed excellent reproducibility of the presented method. A bootstrapping analysis (1 million repetitions) demonstrated that the power of the test, for commonly applied significance levels (0.01, 0.05), amounted to almost 100%. The calculation was performed with H0: both datasets come from the distribution of the control measurements against H1: the difference between the measurements is as observed in the data.

## Discussion

The CBCT examination with a FOV of 5 cm × 5 cm provides detailed information about the cleft side and the corresponding unaffected segment of the maxilla. The new SABG assessment method is useful at every treatment stage and provides excellent repeatability. The null hypothesis was rejected. The maxillary alveolar bone morphology was not the same on the cleft and noncleft sides in UCLP patients after grafting. SABG did not provide good bone morphology, in most cases.

According to the literature, decreasing the CBCT voxel size from 0.4 to 0.25 mm can improve the accuracy of alveolar bone linear measurements. It provides clearer images, easier identification of the alveolar crests, and aligns closer to the gold standard (direct measurements) results [19]. Therefore, the voxel size adopted in this study is adequate. However, examination with a 0.2-mm voxel size provides on average spatial resolution of 0.4 mm. Therefore, it can distinguish objects with a minimum 0.4-mm distance [20]. The spatial resolution is also affected by a scatter lever increasing with FOV size. The recommended reduction of FOV [21] was used in this study, which was smaller than in other papers assessing SABG effectiveness in CLP patients [15–17, 22–25]. The reduction of the FOV size also correlates with an expected reduction of the radiation dose. This approach is in line with ALARA (as low as reasonably achievable) principle. This principle involves maintaining exposures to radiation as far below the dose limits as practical while being consistent with the purpose of the undertaken activity [26]. Some of the studies did not specify the CBCT FOV size [27–29], an integral part of the examination type description.

In cases of healthy periodontal structure, the alveolar bone crest is positioned about 1 mm below the cementoenamel junction. But dehiscences are present if there is a lack of bone coverage on the cervical surface of teeth roots [30]. A drawback to CBCT is a documented underestimation of the bone volume [19, 31]. As a result, a critical point for dehiscence on the CBCT amounting to 2 mm [32] seems justified in the literature. Considering this possibility and the possibility of the lower bone height in the interdental areas than on the root surfaces, the first assessment level was adopted at 3 mm from cementoenamel junction.

Adjustment of the central incisor as the referential tooth seems to provide more adequate assessment of bone architecture. Canines are the tooth most often moved into the grafted area, but central incisors are erupted and routinely aligned before SABG. This fact provides the possibility for reliable bone graft assessments at every treatment stage. Moreover, the central incisors have lower angulation values than the canines in different orthodontic prescriptions [33]. Furthermore, the influence on the measurements of the possible excessive mesial angulation of the canine after orthodontic space closure is eliminated with this approach. The root diameter of central incisors is less than canines. Therefore, the highest score of the horizontal scale was adopted when the thickness of the bone bridge amounts to at least the labiolingual width of the central incisor’s root.

The main aim of the SABG is bone bridge gain [10]. As a result, the unequivocal recognition of the patients without bone bridge appears to be crucial in SABG results classification. Other methods did not take into account the possibility of the narrow bone bridge presence, which was not detected at any adopted measurement level [15–17]. These evaluations appear limited by this omission. In contrast to the bone bridge presence, the bone coverage of the cleft on adjacent roots could not be interpreted as an exclusive result of the SABG procedure. As a result, the new method assessed only the presence and the quality of the bone bridge.

The general assessment of the procedure according to the interval scale provides a possibility for the simple results’ comparison between different studies and surgery modifications. The results obtained on the noncleft side prove that the adopted total score interval in the good results group is adequate.

The most significant factor associated with root resorption in the maxillary arch is the approximation of the incisors against the cortical plate, which could be caused by the camouflage treatment. Also, the orthognathic surgery predisposes teeth to root resorption [34]. A potential root resorption may be a limitation of the presented assessment method due to the frequent skeletal discrepancy in CLP patients. Moreover, patients with complete UCLP and treated with multiband orthodontic appliances have a higher incidence of external apical root resorption on the cleft side maxillary anterior teeth than on the noncleft side [35]. In order to omit this restriction, an assessment modification for severe root resorption cases was presented and used in one patient.

The new method and all of the previously presented horizontal methods [15–17] have excellent intra-rater reproducibility. In the case of inter-rater reproducibility, only the new method and this presented by Garib et al. have excellent reproducibility. This reliability may be explained by a more specific measurement and score criteria. All of these scales provide three-dimensional assessments due to the two-dimensional measurements at different height levels. Therefore, it seems that the vertical analyses presented by Wangsrimongikol et al. [15] and Suomalainen et al. [16] are unnecessary.

A single radiograph is required in case of SABG assessment using the horizontal scales. This requirement is an advantage because two radiographs are not always accessible. Simultaneously, longitudinal measurements could be performed with the CBCT or CT series. This method could also be used in bilateral clefts to assess the SABG results.

Mean follow-up periods in the studies of Wangsrimongkol et al. [15] and Suomalainen et al. [16], amounting to 4.65 and 6.3 months after SABG, may not provide a reliable assessment of the procedure. There is a risk that autogenous grafted bone will not be fully integrated and matured after these periods [12].

The cleft side canines were not fully erupted during the CBCT examinations in two patients operated on before canine eruption. Follow-up periods were 1.17 and 1.25 years, respectively. An improvement of the bone bridge quality can be expected after completion of the eruption process in these patients. The fact that the canines are not always erupted after a year was also noticed by Feichtinger et al. [6]. It may suggest that the time for reliable SABG assessment cannot be determined by the follow-up period. Instead, the determination should be related to the completed teeth eruption process. Lateral incisors or canines should be considered depending on the procedure type.

Among comparative studies, only Garib et al. [17] performed a comparative analysis with the corresponding noncleft areas. These authors obtained significant differences at the cervical and middle measurement levels. No statistically significant differences were found at the apical level. Garib et al. found no bone bridge in 5.56% of the measurement sites on the cleft side. In this study, it was 46.43%. The results may be due to more careful insertion of the bone graft into the cleft area. Also, study group selection might have impacted the results. These authors examined 8 UCLA and 22 complete UCLP patients, and the former group’s presence seems to have influenced the differences between both studies. Patients with UCLA tend to have a partial congenital bone continuity on the palatal side of the alveolus [2]. On the other hand, the above-mentioned study group consisted solely of patients treated with canine mesialization to the cleft area which resulted in contact between the canine and central incisor. This procedure is not possible in the absence of the bone bridge, with considerably disrupted cleft fragments, or when the bone bridge is of general poor quality. Therefore, the results presented refer to a particular group of patients and cannot be used as a general assessment of the surgery [12]. In the study of Suomalainen et al. [16], no bone bridge was found only in 1 site (0.95%). Concomitantly, 27.62% of the measurement sites were not assessed because of the artifacts due to metal fixed orthodontic appliances. Wangsrimongkol et al. [15] described only the method, the study group characteristics, and the raters’ agreement. The assessment results were not presented in their study.

No blinding was used for cleft and noncleft side measurements due to the significant morphologic differences. A limitation of this study was that the raters were only orthodontists. Radiologists, periodontists, oral surgeons, maxillofacial surgeons, and plastic surgeons could also perform alveolar bone measurements. However, orthodontists routinely evaluate the cleft area after grafting for further orthodontic treatment planning.

Even though the size of the study group is limited, assessing SABG outcomes with 3-D x-ray diagnostics in UCLP patients only is consistent with published researches [12]. Patients were qualified according to the eligibility criteria to obtain a general and non-selective assessment of the alveolar bone. Moreover, the analysis of the power calculation demonstrated a reliability of the obtained results.

The follow-up interval was heterogeneous and could have influenced the results, depending on the time required for bone remodeling and bone resorption after SABG. However, Feichtinger et al. [6] have published the longest prospective observation time among all studies that describe 3-D x-ray diagnostics for a SABG treatment outcomes assessment [12]. They conclude that follow-up differences seem to play an insignificant role.

There is a need for further prospective studies to assess the bone bridge architecture in different follow-up periods after grafting. Examining the effect of the bone bridge quality before mesial tooth movement on dehiscence occurrence after space closure in the grafted area should also be considered.

## Conclusions

CBCT provides detailed information about alveolar bone morphology in CLP patients. The new assessment method is useful at every treatment stage and provides excellent repeatability. SABG did not provide good bone morphology, in most cases.

## Electronic supplementary material

ESM 1 (XLSX 13 kb)

ESM 2 (DOCX 19 kb)
